# Licochalcone A as a Potential Anti-*Toxoplasma* Agent: A Target Identification and Pharmacokinetic Study

**DOI:** 10.3390/biom16030410

**Published:** 2026-03-10

**Authors:** Bing Li, Zexin Tao, Yichen Jing, Yubin Bai, Weiwei Wang, Bintao Zhai, A. M. Abd El-Aty, Chao Zhang, Jiyu Zhang, Fangdi Hu

**Affiliations:** 1 School of Pharmacy, Lanzhou University, Lanzhou 730000, China; libing@caas.cn (B.L.); taozx2023@lzu.edu.cn (Z.T.); jingych2023@lzu.edu.cn (Y.J.); 2 Lanzhou Institute of Husbandry and Pharmaceutical Sciences, Chinese Academy of Agricultural Sciences, Lanzhou 730050, China; baiyubin@caas.cn (Y.B.); wangweiwei@caas.cn (W.W.); zhaibintao@caas.cn (B.Z.); zc196010@sjtu.edu.cn (C.Z.); 3 Department of Pharmacology, Faculty of Veterinary Medicine, Cairo University, Giza 12211, Egypt; abdelaty44@hotmail.com; 4 Department of Medical Pharmacology, Medical Faculty, Ataturk University, 25240 Erzurum, Turkey

**Keywords:** licochalcone A, *Toxoplasma gondii*, *Tg*MORN1, SPR, AI, pharmacokinetic

## Abstract

Toxoplasmosis is a zoonotic disease with limited therapeutic options, which are further hampered by significant toxicity and suboptimal efficacy. Effective interventions for chronic infection remain insufficient, and thus, natural product-derived drug screening remains a key focus in anti-*Toxoplasma* research. Licochalcone A (Lico A), a major bioactive compound isolated from *Glycyrrhiza uralensis*, exhibits potent activity against *Toxoplasma* tachyzoites. However, systematic studies of its targets, pharmacokinetics, and efficacy are lacking, hindering its development as an anti-*Toxoplasma* candidate drug. In this study, we used SPR-MS to identify 33 high-affinity target proteins (affinity score > 1000). Furthermore, an AI-driven multidimensional analysis identified a cluster of five proteins (*Tg*MORN1, D3XD37, ABCB2, MIC15, and IDH), with *Tg*MORN1 yielding the highest composite score. RNAi experiments confirmed *Tg*MORN1 as a key target, as its silencing attenuated the anti-proliferative effect of Lico A. Western blotting, NanoDSF, and SPR supported direct binding between Lico A and *Tg*MORN1, suggesting that Lico A modulates *Tg*MORN1 thermal stability through residues S168 and D203, with high species specificity. Pharmacokinetic evaluation revealed that Lico A had favorable absorption and blood–brain barrier permeability, supporting its potential utility in treating brain disease. In vitro assays showed that Lico A effectively inhibited *Toxoplasma gondii* brain cyst formation. Collectively, these findings support Lico A as a promising candidate for the treatment of toxoplasmosis.

## 1. Introduction

Toxoplasmosis is a globally distributed zoonosis that poses a particularly severe threat to pregnant women, fetuses, and individuals with a weakened immune system [1]. *Toxoplasma gondii* forms tissue cysts in the brain and causes a variety of chronic diseases, such as schizophrenia [2], bipolar disorder [3], and Alzheimer’s disease [4]. Therefore, controlling the spread of toxoplasmosis is critically important. Currently, the standard treatment for acute toxoplasmosis in clinical practice typically involves triple therapy consisting of sulfonamide (such as sulfadiazine), pyrimethamine, and folinic acid (calcium folinate) supplementation [2]. However, there remains a significant lack of effective therapeutic options for chronic *Toxoplasma* infections. Therefore, it is necessary to develop more effective drugs to treat toxoplasmosis and reduce infections. In recent decades, the emergence of drug resistance, environmental pollution, and other issues related to commercial antiparasitic agents has significantly hindered their efficacy [5]. Consequently, the development of eco-friendly alternative agents derived from sustainable natural products has become increasingly desirable and necessary for both human health and agricultural applications.

Lico A, a flavonoid extracted from the root of the licorice plant (a member of the legume family), exhibits antimalarial and antileishmaniasis activities [6,7]. Furthermore, research has demonstrated its antioxidant, antibacterial, antiangiogenic, and antitumor properties [8]. Our previous studies indicated that Lico A has significant anti-*Toxoplasma* effects with low cytotoxicity [6]. In vitro experiments have shown that Lico A can prevent *Toxoplasma gondii* from invading host cells and inhibit their intracellular replication [6]. In vivo studies revealed a survival rate of up to 100% in infected mice treated with Lico A [6]. Transmission electron microscopy revealed alterations in the ultrastructures of the parasites following treatment, whereas Nile red staining suggested a relationship between the effects of Lico A and lipid metabolism [6]. However, the potential target sites of Lico A remain unclear, and its potential as a drug for the treatment of toxoplasmosis also requires further research.

In this study, we used the RH strain of *T. gondii* as the model and employed surface plasmon resonance (SPR) [9] and an artificial intelligence-driven scoring framework (AI) [10,11,12,13] to identify the potential insecticidal targets of Lico A and conduct verification [14,15,16]. Additionally, pharmacokinetic evaluations and in vitro activity assays were conducted to assess its anti-*T. gondii* efficacy, particularly against chronic infection. This study aims to elucidate the potential of Lico A as a candidate for the treatment of toxoplasmosis.

## 2. Materials and Methods

### 2.1. Chemicals and Reagents

The *T*. *gondii* RH strain and PRU strain were obtained from Lanzhou Veterinary Research Institute, Chinese Academy of Agricultural Sciences. BL21 (DE3) competent *Escherichia coli* were acquired from Thermo Fisher Scientific (Waltham, MA, USA), item number EC0114; human foreskin fibroblasts (HFFs) were acquired from the American Type Culture Collection (ATCC, Manassas, VA, USA), item number CRL-1634. Lico A was acquired from Med Chemexpress (MCE, Monmouth Junction, NJ, USA), item numberHY-N0372.Centrifuge (Thermo Fisher, Waltham, MA, USA),SPR Biochip Analysis System-1 (Berthold, Bad Wildbad, Germany),SPR Unlabeled Interaction Analyzer-2 (PLEXERA LLC, Seattle, WA, USA), Chip microarray printer (BioDot Corporation, Irvine, CA, USA), Gel imaging system (Tanon, Shanghai, China), Fluorescence quantitative PCR (Thermo Lifetech ABI, Waltham, MA, USA), Cell counter (Millipore, Billerica, MA, USA), Protein Stability Analyzer (NanoTemper, Munich, Germany), Flow cytometer (Beckman Coulter, Pasadena, CA, USA), HPLC-MS/MS (AB Sciex, Boston, MA, USA). The SPF-grade BALB/c female mice used were purchased from Lanzhou Veterinary Research Institute, Chinese Academy of Agricultural Sciences.The license number is SYXK (Gan) 2020-0002, and the animal qualification certificate number is 0002094. The laboratory was raised in the SPF-level standardized laboratory animal room of the Lanzhou Institute of Animal Science and Veterinary Medicine, Chinese Academy of Agricultural Sciences, and the laboratory animal use license number is SYXK (Gan) 2019-0002. The animal experiments were approved by the Animal Ethics Committee of Lanzhou Institute of Husbandry and Pharmaceutical Sciences of CAAS (Approval protocol number:2024-011; Approval date: 30 April 2024). The experimental process complied with the “Regulations of Lanzhou Institute of Animal Science, Chinese Academy of Agricultural Sciences”.

### 2.2. Cells and Toxoplasma gondii

Human foreskin fibroblasts (HFFs) were cultured in phenol red-containing DMEM supplemented with 10% FBS, 1% GlutaMAX, 1% nonessential amino acids (NEAA), and 1 mM sodium pyruvate. *T. gondii* tachyzoites were inoculated onto confluent cell monolayers for passaging. Tachyzoites of the *Toxoplasma gondii* RH strain were briefly centrifuged and then resuspended in 120 μL of PBS containing protease inhibitors. The suspension was thoroughly vortexed to ensure complete resuspension, followed by the addition of lysis buffer. The mixture was incubated at 4 °C and centrifuged at 16,000× *g* for 10 min. The protein concentration was measured via a BCA protein assay kit, and the samples were adjusted to a final concentration of 200 μg/mL with freshly prepared buffer.

### 2.3. Target Protein Capture and Identification by SPR-MS 

Candidate protein targets of Lico A were enriched and identified through an experimental approach employing surface plasmon resonance coupled with mass spectrometry (SPR-MS) [17]. Biological chips (Better Ways INC, Guangzhou, China) were fabricated via high-throughput array printing, dried, photocrosslinked, washed, quality checked, and stored at −20 °C. During analysis, the chip surfaces were scanned, and the resonance angles were optimized. Lico A was dissolved in DMSO to prepare a 10 mM solution, which was then covalently immobilized onto the surface of the sensor chip. *T. gondii* cells were centrifuged, resuspended in PBS supplemented with protease inhibitors, lysed on ice for 10 min, and then centrifuged at 16,000× *g* for 10 min at 4 °C. Protein concentrations were measured with a BCA assay and adjusted as needed. Chips were installed in the SPR system, regenerated with Gly·HCl (pH 2.0), blocked with BSA, and baseline adjusted. PBS was added at 2 μL/s for 260 s to establish equilibrium. *T. gondii* lysates were circulated over the chip for 260 s to record signals, followed by washing. Protein interactions with the immobilized ligand were monitored in real time by SPR, which quantitatively recorded the binding affinities. The proteins bound to the chip surface subsequently underwent on-chip enzymatic digestion, and the resulting peptides were analyzed via liquid chromatography–tandem mass spectrometry (LC–MS/MS) to determine the identities of the interacting proteins. The data were searched against the UniProtKB/Swiss-Prot database via Mascot in Proteome Discoverer. The functional characterization of the identified candidate targets was performed via bioinformatic annotation. Each protein was categorized into molecular function, cellular component, or biological process categories based on the Gene Ontology (GO) database. Simultaneously, the candidate proteins were mapped to the Kyoto Encyclopedia of Genes and Genomes (KEGG) pathway database to identify significantly enriched signaling and metabolic pathways.

### 2.4. Multidimensional Target Scoring Model

To prioritize the most plausible key targets from the candidate protein list, we constructed a multidimensional scoring framework integrating intrinsic protein features, pharmacological semantic relevance, and protein–protein interaction (PPI) network topology. The model takes candidate proteins identified by surface plasmon resonance mass spectrometry (SPR-MS), functional annotations (GO and KEGG), and the chemical structural information of Lico A as inputs. A feature representation module constructs diverse vector embeddings for each protein, encompassing sequence- and structure-based attributes, functional annotations, and network topological properties. All computational analyses were conducted in Python 3.10 using PyTorch, Scikit-learn, NetworkX, and RDKit, with random seeds fixed (seed = 42) to ensure reproducibility. For intrinsic feature representation, protein sequence embeddings were generated using a pretrained transformer-based protein language model (ESM-2, 650M parameters; embedding dimension 1280), followed by principal component analysis retaining 95% variance (reduced to 256 dimensions). To estimate drug-target-like characteristics, a one-class support vector machine (OC-SVM; RBF kernel, ν = 0.1) was trained using approximately 1200 curated drug targets from Drug Bank as positive reference data. The pharmacological relevance module quantified semantic similarity between each protein’s functional annotation profile and the known biological effects of Lico A. Functional descriptors derived from GO and KEGG annotations were encoded using a biomedical language model (BioBERT, 768-dimensional embeddings). For system-level evaluation, a high-confidence *T. gondii* PPI network was constructed using STRING (v11.5; interaction confidence ≥ 0.7). Binding pockets were predicted using Fpocket (minimum volume 200 Å^3^), and molecular docking was performed using AutoDock Vina -v4.2.6; exhaustiveness = 16; grid size 20 × 20 × 20 Å; energy threshold −6.0 kcal/mol). All three primary dimension scores were min–max normalized to ensure comparability. The final integrated score was computed as a weighted aggregation:

Final Score = 0.45 × Pharmacological Relevance + 0.30 × Network Score + 0.25 × Feature Score. Weights were determined through grid search optimization (step size 0.05) to maximize ranking stability and separation among top candidates. Through weighted integration of these features, the model enables multidimensional prioritization of potential targets, identifying the most promising key proteins for further experimental validation.

### 2.5. Protein–Protein Interaction Network Analysis

Building on the prioritized targets, we extracted validated and predicted protein–protein interactions (PPIs) from STRING to construct a protein–protein interaction (PPI) network, which revealed the complex relationships among candidate proteins. Topological analysis via Cytoscape-3.10.4 identified key modules and hub nodes, highlighting clusters that may indicate functional pathways. By integrating multidimensional scores with network topology, we refined potential targets, focusing on those with significant biological relevance for further validation.

### 2.6. Design and Synthesis of dsRNA

Double-stranded RNA (dsRNA) was synthesized by Yangling Tianrunke Bio. (China), according to the sequence of the target gene NADH dehydrogenase subunit 2 (Psoroptes cuniculi) (NCBI, NC_2364249.2) (*Tg*MORN1). The forward primer was 50-GGAAAGGGACUCUGAUUUATT-30, and the reverse primer was 50-UAAAUCAGAGUCCCUUUCCTT-30. dsRNA was quantified via a NanoDrop spectrophotometer (Thermo Fischer Scientific, Waltham, MA, USA). Finally, the dsRNA was diluted in normal saline (Solarbio, Beijing, China) to a final concentration of 500 nM and stored at 20 °C.

### 2.7. MORN1 Gene Knockdown Assays

*T. gondii* tachyzoites were maintained in HFF monolayers with DMEM containing 3% FBS under the conditions described in Section 2.2. After harvesting, the parasites were washed twice with sterile distilled water and resuspended in 24-well plates. For the knockdown assay, each well received 500 µL of solution containing 500 nM *Tg*MORN1-dsRNA mixed with a commercial transfection reagent (Invigentech, Irvine, CA, USA). The parasites were incubated at 25 ± 1 °C and ~75% relative humidity for 48 h. The control group was treated with an equal volume (500 µL) of distilled water under identical conditions. Following incubation, samples were collected for RNA extraction and subsequent expression analysis via RT–qPCR. All the treatments were performed in triplicate to ensure reproducibility [9].

### 2.8. Quantification of Gene Expression by RT–qPCR

The dsRNA-treated *Toxoplasma gondii* and untreated controls were rinsed twice with PBS before RNA isolation. Total RNA was extracted using the RNeasy Mini Kit (Qiagen, Germany) according to the manufacturer’s instructions. The RNA concentration and purity were assessed with a NanoDrop spectrophotometer, and sample integrity was checked via agarose gel electrophoresis. Approximately 1 µg of RNA from each sample was reverse-transcribed into cDNA via the QuantiTect Reverse Transcription Kit (Qiagen, Germany), which includes a genomic DNA removal step.

Quantitative PCR was carried out in a 20 µL reaction volume containing TB Green Premix Ex Taq II (Takara, Japan), specific primers, and a cDNA template on a QuantStudio 6 Flex Real-Time PCR System (Applied Biosystems, USA). The thermal program consisted of initial denaturation at 95 °C for 30 s, followed by 40 cycles at 95 °C for 5 s and 60 °C for 30 s. A melting curve analysis was performed at the end of the reaction to confirm amplification specificity. The primers for MORN1 were forward 5′-TGGGAAATGGGCCGAATCAA-3′ and reverse 5′-CTTTTCCGTGGCGCTTATCG-3′, and *α-tubulin* was used as the internal reference gen [18]. Relative transcript abundance was determined via the 2^−ΔΔCt approach [19]. Each reaction was run in triplicate, and all data collection and reporting followed the MIQE 2.0 recommendations [20].

### 2.9. Protein Expression and Purification

To construct the expression plasmid, the full-length *Tg*MORN1 gene was synthesized with a C-terminal His_6_-tag and cloned into the pT7CFE1-RADA vector, and the construct was subsequently transformed into *Escherichia coli* BL21 (DE3) for protein expression. The bacteria were grown at 37 °C in Terrific Broth containing 30 mg/L kanamycin to an optical density of 0.6–0.8 at 600 nm, induced at 15 °C with 0.5 mM isopropyl-β-d-thiogalactopyranoside (IPTG) and further grown overnight at 15 °C. The bacteria were collected by centrifugation, and the pellets obtained were immediately used for subsequent steps. The pellets were suspended in lysis buffer (containing protease inhibitors, 50 mM Tris, 300 mM NaCl, and 10% glycerol, pH 8.0) and sonicated in an ultrasonic bath on ice. Cell lysis was performed, generating a crude protein sample. The lysed protein sample was diluted 5-fold with balancing buffer (containing 50 mM Tris, 300 mM NaCl, and 10% glycerol, pH 8.0) and incubated with nickel–Sepharose beads (CWBIO). The protein was eluted with different concentrations of imidazole (50 and 500 mM), and the absorption peaks were detected. The subsequent sample was eluted with the absorption peak-indicated concentration of imidazole.

### 2.10. Antibody Preparation

Specific antibodies against the *Tg*MORN1 protein were prepared by immunizing six New Zealand White rabbits. The immunization protocol involved an initial injection (*Tg*MORN1 antigen + complete Freund’s adjuvant) followed by two booster injections (incomplete Freund’s adjuvant). After blood collection, immune serum titers were evaluated via ELISA. The serum was subsequently affinity-purified via a *Tg*MORN1-coupled affinity chromatography column to obtain high-purity antibodies. The purity and integrity of the purified antibodies were verified via SDS–PAGE, and their titers were redetermined via ELISA.

### 2.11. Western Blot

Western blot analysis was conducted to assess protein expression in *Toxoplasma gondii* tachyzoites after Lico A treatment. Parasites were washed twice with PBS, lysed in RIPA buffer, and sonicated on ice. The lysates were clarified via centrifugation (12,000 rpm, 10 min, 4 °C), and protein concentrations were determined via the BCA method. Equalized samples were mixed with loading buffer, boiled for 5 min at 100 °C, and separated on 15% SDS–PAGE gels. Proteins were transferred to PVDF membranes (0.20 µm) at 110 V for 1 h. After blocking with 5% BSA, the membranes were incubated overnight at 4 °C with primary antibodies against MORN1 (1:1000) and α-tubulin (1:2000). After three washes with TBST, HRP-conjugated secondary antibodies (1:2000) were applied for 2 h at room temperature. Bands were visualized by enhanced chemiluminescence (ECL) (Millipore) and imaged with a Bio-Rad ChemiDoc system. *α-Tubulin* served as the internal control. All experiments were performed independently in triplicate, following a previously reported method [21].

### 2.12. Nanodifferential Scanning Fluorimetry (NanoDSF)

NanoDSF, with its rapid and sensitive characteristics, has become an ideal method for investigating protein–compound binding and its impact on protein thermal stability [22]. Temperature curves were acquired via a Nanotemper Prometheus NT.48 fluorimeter (Nanotemper, version 2.1.2) to analyze protein–compound binding. A *Tg*MORN1 protein sample diluted 1:1 with buffer served as a blank control. Concurrently, the experimental group consisted of *Tg*MORN1 protein mixed with an equal volume of 10 μM Lico A (final protein concentration of 0.2–0.5 mg/mL). The samples were heated from 20 °C to 95 °C at a rate of 1 °C/min. The protein samples were excited at 280 nm, and changes in fluorescence intensity were monitored at 330 nm and 350 nm. The fluorescence ratio at 350 nm to 330 nm and its first derivative were recorded and calculated as a function of temperature.

### 2.13. Binding Affinity Analysis

For affinity analysis between small molecules and proteins, a chemically modified label-free photocrosslinking SPR chip was utilized. Small molecules were covalently immobilized via UV photocrosslinking to preserve their native conformation. Positive controls (rapamycin–FKBP12 and biotin–streptavidin systems) and solvent/blank controls (DMSO and PBS) were included to validate chip performance. After printing and UV photocrosslinking of the candidate compounds, the ELP2 protein was injected into the SPR system for association and dissociation measurements, followed by chip regeneration. The raw data were preprocessed and fitted to a 1:1 Langmuir binding model to calculate the association rate constant (Kon), dissociation rate constant (Koff), and equilibrium dissociation constant (KD), thereby quantifying the affinity characteristics [23].

### 2.14. Ligand Preparation and Protein Docking Analysis 

Lico A was prepared via the LigPrep module in Schrödinger to generate 3D conformations and optimize the geometry under the OPLS4 force field [23]. Protonation states and tautomers were predicted with Epik, and ADME properties were evaluated via QikProp. Conformers with high-energy (>20 kcal/mol) or unstable groups were excluded, and the lowest-energy structures were retained for docking. The MORN1 protein sequence was retrieved from UniProtKB and modeled via SWISS-MODEL with templates showing > 40% identity and > 85% coverage. Structural refinement was performed with the Protein Preparation Wizard, followed by Ramachandran validation (>95% residues in favored or allowed regions). Potential binding pockets were predicted via SiteMap, and the top-ranked site (SiteScore > 0.90, DScore > 0.98) was selected for grid generation (10 × 10 × 10 Å^3^). Docking was carried out with Glide in XP mode, treating the ligand as flexible and the protein as rigid. The best-ranked poses were analyzed for hydrogen bonding, hydrophobic contacts, and π–π interactions. The top five conformations were further evaluated via MM-GBSA to estimate the binding free energy (ΔG_bind).

### 2.15. Cell-Free In Situ Expression Chip Preparation and Binding Analysis

For affinity analysis, cell-free in situ expression chips were prepared. Plasmids encoding six MORN1 mutants (R39A, T52A, S168A, D203A, D263A) and wild-type MORN1 were cloned and inserted into the pT7CFE1-RADA vector and verified by Sanger sequencing. These plasmids were array-printed onto RADA chips (with four technical replicates per sample) and UV-crosslinked to immobilize the DNA templates. In situ protein expression was subsequently performed via a cell-free human in vitro translation (IVT) system in a confined microenvironment (37 °C, 100% humidity), followed by covalent protein fixation with a BS3 crosslinker. The chips were then washed, nitrogen-dried, and packaged. SPR monitoring confirmed that all expressed proteins were present at nanogram levels or higher, making them suitable for subsequent affinity analysis.

### 2.16. Protein Homology Analysis and Pharmacological Cross-Reactivity Prediction

The homology and potential pharmacological cross-reactivity between the target protein and human MORN-like proteins were evaluated. First, the target protein sequence was retrieved from UniProt and aligned using local BLAST+ (v2.13.0) to identify human homologs, with filters applied for an E value < 1 × 10^−5^, sequence coverage ≥ 80%, and homology ≥ 30%. Multiple sequence alignment was subsequently performed using Clustal Omega (v1.2.4, default gap penalties), with a focus on the conservation of key residues near the binding pocket (e.g., S168, D203). Finally, a neighbor-joining phylogenetic tree was constructed via MEGA X to predict drug selectivity and off-target risk.

### 2.17. Pharmacokinetic (PK) Study 

The animal study was conducted in strict accordance with the National Medical Products Administration (NMPA) Good Laboratory Practice (GLP) guidelines at the SPF-class housing facility of the Lanzhou Institute of Husbandry and Pharmaceutical Sciences, Chinese Academy of Agricultural Sciences. Sprague Dawley (SD) rats were obtained from Lanzhou Veterinary Research Institute, Chinese Academy of Agricultural Sciences. (Lanzhou, China). Specific pathogen-free (SPF)-grade female BALB/c mice (n = 9 per group; weight: 20–21 g; age: 7–12 weeks) were administered Lico A at doses of 300 mg/kg and 150 mg/kg [24] (solubilized in 13% Tween-80) via oral gavage (i.g.) and intraperitoneal injection (i.p.), respectively. Blood samples were collected at specified intervals post-administration (0.25, 0.5, 1, 2, 4, 8, 12, and 24 h), followed by centrifugation at 4000 rpm (Thermo Scientific Sorvall ST 16R Centrifuge, Osterode, Germany) and 4 °C for 10 min. The supernatant plasma was stored at −20 °C for subsequent analysis.

Analysis was performed on a Triple Quad 5500 high-performance liquid chromatography–mass spectrometry system (SCIEX, USA) (LC–MS/MS). The samples were separated on a Waters XBridge C18 column (3.5 μm, 2.1 × 50 mm) (Waters, USA). The column temperature was maintained at 30 °C, and the mobile phase consisted of water with 0.1% formic acid (*v/v*, buffer A) and methanol with 0.1% formic acid (*v/v*, buffer B). The elution was conducted according to a 2.5 min gradient program (0–0.5 min, 60% buffer B; 0.5–1.2 min, 98% buffer B; 1.2–2.0 min, 98% buffer B; 2.01–2.5 min, 60% buffer B), with the flow rate set at 0.5 mL/min and the injection volume at 2 μL. Mass spectrometry was performed in positive MRM mode, targeting the quantitative ion pairs of Lico A (*m/z* 339.2/121.2 and 953.3→275.1, respectively). Glibenclamide was used as the internal standard (IS), with a quantitative ion pair of 494.2/369.1. The plasma concentration exhibited a linear response within the range of 5–5000 ng/mL. The pharmacokinetic parameters were calculated via a noncompartmental model in Phoenix 8.3 software.

### 2.18. Brain Distribution Study 

Female BALB/c mice (n = 9 per group, weights: 20–21 g, age: 7–8 weeks, SPF grade) were administered Lico A at a dose of 300 mg/kg (dissolved in 13% Tween-80) via oral gavage (i.g.). Blood samples and brain tissues were collected at specified intervals post-administration (0.5 h, 1 h, and 8 h). Blood sample processing was performed as described in Section 2.16. The brain tissues were rinsed with precooled (2–8 °C) physiological saline, blotted dry with filter paper, homogenized in 20% methanol–water (1:5, w/v), and then stored at −20 °C for subsequent analysis.

LC–MS/MS analysis was performed as described in Section 2.17, with the exception that the brain tissue samples were separated on a Waters Xselect HSS T3 column (3.5 μm, 2.1 × 50 mm), with an injection volume of 10 μL. The plasma concentration exhibited a linear response within the range of 1–1000 ng/mL. The pharmacokinetic parameters were calculated via a noncompartmental model in Phoenix 8.3 software, and the brain-to-plasma free drug ratio (Kp) was calculated.

### 2.19. T. gondii PRU Tachyzoite Activity 

Monolayer Vero cells in 12-well plates were infected with PRU, inoculated with 1 × 10^4^ tachyzoites per well, and cultured in 1% FBS medium for 8 h. Then, DMEM containing the corresponding concentration (1 μg/mL, 2 μg/mL, or 4 μg/mL) of Lico A was added, and two wells were set for each concentration. After incubation for 24, 48, and 72 h, the number of vesicles (PVs) of the PRU strains was observed and counted under a 40× objective microscope, with 3 visual fields being randomly observed in each well. Under a microscope, the tachyzoites of *Toxoplasma gondii* in the control group almost completely overflowed, and the cells were completely broken. The plaques were fixed with 4% paraformaldehyde, incubated at room temperature for 10 min, and dyed with crystal violet staining solution for 10 min [25]. After drying at room temperature, the number and size of the plaques were observed and analyzed with a microscope.

### 2.20. Flow Cytometry Assay 

Vero cells were inoculated in 25 cm^2^ culture flasks and infected with tachyzoites of the PRU strain. 24 h after infection, the samples were treated with Lico A (1 μg/mL, 2 μg/mL, or 4 μg/mL), and the control group was treated without medication. Approximately 1 × 10^6^ *T. gondii* tachyzoites were extracted 24 h after incubation and centrifuged at 1500× *g* for 10 min. The samples were washed with PBS, suspended in 100 µL of binding buffer containing 5 µL of annexin V-PE and 5 µL of FITC dye and incubated at room temperature in the dark for 20 min. The tachyzoite survival rate of each group was measured via flow cytometry. The experiment was independently repeated three times [25].

### 2.21. Cyst Formation of the T. gondii PRU Strain

The 12-well plates were infected with alkaline-pretreated PRU tachyzoites and cultured under conditions of blyzoite induction (RPMI 1640 medium with 50 mM HEPES and 1% fetal bovine serum at pH 8.2) for 3 d. Subsequently, DMEM containing Lico A (2 μg/mL, 4 μg/mL) was used as a control. The size and shape of the cysts were observed and recorded by a Laser Scanning Confocal Microscope after they were incubated with the drug mixture two times for 48 h and DBA staining.

### 2.22. Sequence Alignment and Data Statistics

Homology analysis of protein sequences was performed via the BLAST algorithm, with homologous sequences obtained from the UniProt database. Sequence alignment was conducted via Clustal Omega-1.2.4 software. Data from each experimental stage were analyzed via GraphPad Prism (version 9.1). The results are expressed as the means ± standard deviations (SDs). Statistical significance was assessed via two-tailed Student’s t test or one-way ANOVA, with a minimum of three replicates for each measurement, unless otherwise stated.

### 2.23. Statistical Analysis

Data statistical analysis was conducted using SPSS 19.0 software (SPSS Inc., Chicago, IL, USA) and GraphPad Prism 6 (San Diego, CA, USA). A *p* value less than 0.05 indicated a significant difference, while a *p* value less than 0.01 indicated an extremely significant difference.

## 3. Results

### 3.1. Screening of Lico A’s Anti-T. gondii Target Proteins

**Figure 1:**
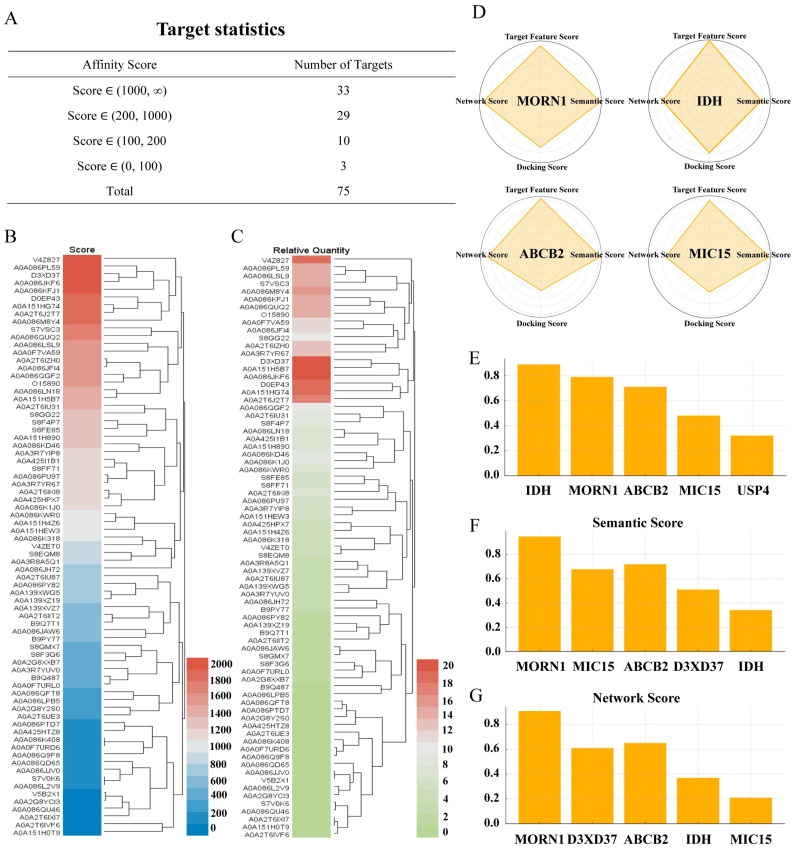
Screening proteins with anti-*Toxoplasma* target characteristics via SPR-MS and a multidimensional scoring model. (**A**) Summary table presenting the target protein proteomics results, highlighting a positive correlation between the affinity score and the compound-protein binding capacity. (**B**) Heatmap depicting the scoring results from the target protein proteomics analysis. (**C**) Heatmap illustrating the enrichment abundance from the analysis chip. (**D**) The final comprehensive score shows that MORN1 has the highest score and is predicted to be the most likely key target. As an enzyme protein involved in lipid metabolism. IDH performs well in terms of structural similarity and comparison with known targets but has a weak correlation with the pharmacological mechanism of Lico A. Although ABCB2 may bind Lico A in molecular docking, its criticality and semantic association in parasitic physiology are limited, resulting in a relatively low score. MIC15 ranked fourth in terms of overall score due to its relatively weak structural compatibility. (**E**) The target feature scoring module evaluates the functional similarity between candidate proteins and known drug targets. (**F**) The pharmacological relevance scoring module estimates the semantic correlation between candidate proteins and the known mechanisms of action of Lico A. (**G**) The network-based scoring module assesses the regulatory potential of candidate proteins on the basis of centrality indices within the protein–protein interaction (PPI) network.

To analyze the pharmacological mechanism of Lico A in *Toxoplasma gondii* in detail, this study adopted surface plasmon resonance–mass spectrometry-based target protein capture technology. The target proteomics method of SPR-MS was used to systematically screen and identify potential binding proteins in the whole proteome of this parasite. The analysis results revealed that Lico A could bind to 75 proteins in the *Toxoplasma gondii* proteome, among which 33 proteins exhibited high binding affinity (Figure 1A). Two-dimensional graphs of the captured target proteins were constructed using confidence levels and relative expression abundances, and multiple proteins were found to be in the ranges of high expression and high pharmacodynamic correlation. In target capture analysis, the score is positively correlated with affinity. The relative quantity is a value obtained from mass spectrometry measurements and represents the relative proportion of each captured protein on the chip. Scoring heatmap analysis (Figure 1B) and relative abundance heatmap analysis (Figure 1C) were subsequently performed on the target proteins.

To refine the identification of key therapeutic targets of Lico A from the 33 specific candidate binding proteins captured by SPR-MS, a multidimensional scoring strategy was employed (Figure S1). This approach combines three complementary modules: target feature scoring, pharmacological relevance (semantic) scoring, and network scoring, which incorporate protein–protein interaction (PPI) topological features. Through this integrated analysis, *Tg*MORN1, D3XD37, ABCB2, IDH, and MIC15 emerged as prominent nodes within the PPI network (Figure 1D). Among them, *Tg*MORN1, a parasite-specific basal complex protein, was predicted to interact with multiple cell proliferation-related proteins and displayed a highly clustered state in the network. After normalization and integration across dimensions, *Tg*MORN1 achieved the highest overall score, followed by IDH, ABCB2, and MIC15 (Figure 1E-G). *Tg*MORN1 was significantly enriched in the SPR-MS results, with an affinity score of 1670.13 (out of 2000) and a relative abundance of 15.70%. Taken together, these results support *Tg*MORN1 as the most likely key target of Lico A and the focus of subsequent mechanistic studies.

### 3.2. MORN1 Target Validation via Rnai Technology

**Figure 2:**
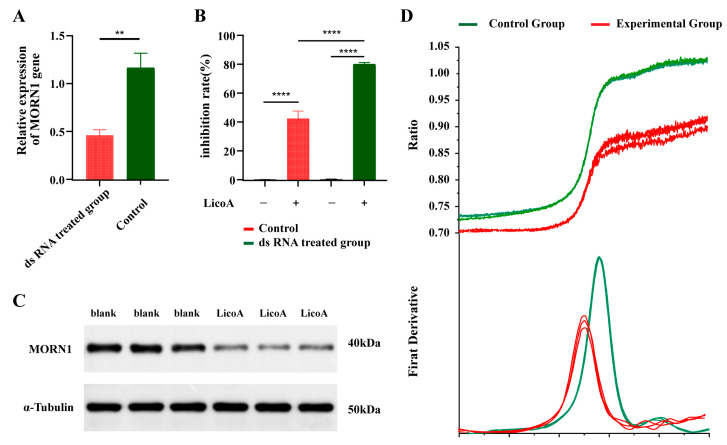
Validation of *Tg*MORN1 as the molecular target of Lico A in *Toxoplasma gondii* tachyzoites. Lico A directly interacts with *Tg*MORN1 and alters its expression and stability. (**A**) Transcription levels of the *Tg*MORN1 gene in *Toxoplasma gondii* before and after dsRNA treatment. (**B**) Rate of Lico A inhibition of *Toxoplasma gondii* before and after dsRNA treatment. (**C**) Western blot analysis of *Tg*MORN1 in tachyzoites. Compared to the blank control, treatment with 4 μg/mL Lico A significantly reduced the *Tg*MORN1 protein level, indicating the downregulation of *Tg*MORN1 expression (Original images can be found in Figure S6). (**D**) Thermal stability of *Tg*MORN1 as assessed by NanoDSF. The addition of 10 μM Lico A induced a clear shift in the melting curve, reducing the melting temperature (Tm) from 48.1 °C to 44.0 °C, demonstrating the direct interaction of Lico A with *Tg*MORN1 and decreased protein stability (** significant at *p* < 0.01; **** significant at *p* < 0.001).

Tachyzoites were incubated with dsRNA targeting *Tg*MORN1 for 48 h via the soaking method, and transcriptional levels were quantified via RT–qPCR. Compared to that in the control group, *Tg*MORN1 mRNA expression was markedly lower in the silenced parasites (*p* < 0.01), with an average decrease of approximately 70.00% (Figure 2A), indicating that *Tg*MORN1-knockdown strains were successfully established and could be employed for subsequent target validation studies. These strains, together with control parasites, were then treated with Lico A to assess its inhibitory effect on *Toxoplasma gondii* tachyzoite proliferation. In the control group, Lico A suppressed tachyzoite growth by 80.58%, whereas in *Tg*MORN1-silenced parasites, the inhibition rate decreased significantly to 43.07% (*p* < 0.001) (Figure 2B).

### 3.3. Expression and Purification of TgMORN1 and Production of Polyclonal Antibodies

The recombinant *Tg*MORN1 protein expressed from the constructed plasmid was soluble and could be purified to high homogeneity. SDS–PAGE together with Western blot analysis revealed a clear band at the expected molecular weight. Induction at 15 °C with 0.5 mM IPTG markedly increased the proportion of soluble protein. The recombinant product was then purified by Ni^2+^ affinity chromatography, yielding a highly soluble and pure protein suitable for immunization (Table S1).

This purified *Tg*MORN1 protein served as the antigen for the immunization of New Zealand white rabbits, involving one primary injection and three boosters. ELISAs indicated that the final serum titer reached 1:128000. After affinity purification and dialysis, two batches of polyclonal antibodies were obtained at 1 mg/mL (2.1 mL and 1.4 mL). SDS-PAGE analysis confirmed their high purity (Figure S2).

### 3.4. Approbation of Intermolecular Interactions Between Lico A and TgMORN1 Determined by WB, NanoDSF, and SPR

To investigate the effect of Lico A on *Tg*MORN1, Western blot (WB) analysis was performed in the control and Lico A-treated groups. As shown in Figure 2C, compared to the blank control, exposure to 4 μg/mL Lico A resulted in a pronounced reduction in the *Tg*MORN1 protein level, which demonstrates that Lico A downregulates *Tg*MORN1 expression.

NanoDSF analysis revealed a clear shift in the melting curve after the addition of 10 µM Lico A. The melting temperature (Tm) of *Tg*MORN1 decreased from 48.1 °C to 44.0 °C in the presence of Lico A, indicating the protein’s reduced stability (Figure 2D).

**Figure 3:**
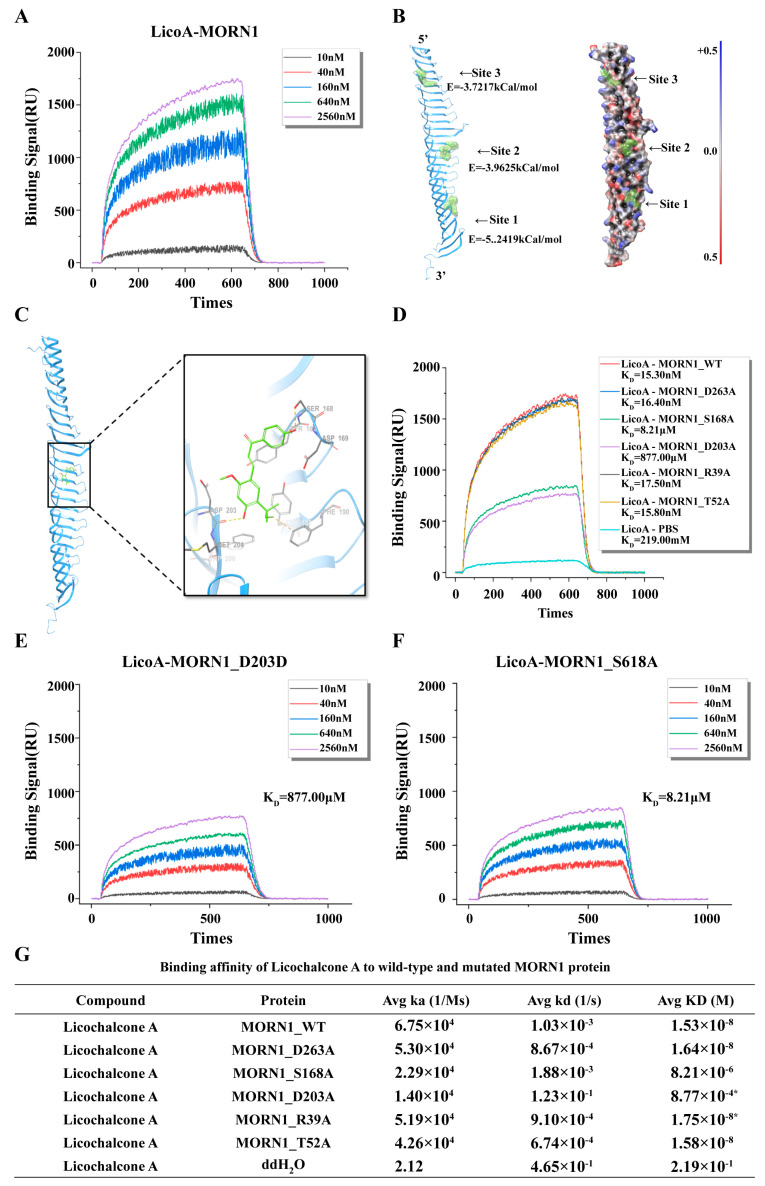
Interaction verification and binding site analysis. (**A**) SPR binding curve illustrating the interaction between Lico A and MORN1 across five logarithmic concentration gradients. (**B**) Illustration of three potential binding regions for Lico A on MORN1 through secondary structure representation and spatial structure depiction. The value in parentheses indicates the binding free energy of the compound at that site. (**C**) Schematic representation of bonding interactions between Lico A and nearby amino acid residues at the binding site, with purple arrows denoting hydrogen bonds, yellow arrows denoting van der Waals forces, and green arrows denoting hydrophobic interactions. Summary table of affinity constants. (**D**) The SPR assessment of affinity for wild-type and five point-mutant variants of MORN1, comparing affinity constants (KD). (**E**,**F**) Concentration gradient binding curves for licochalcone A with wild-type MORN1 (A) the S168A mutant (**E**) and the D203A mutant (**F**). (**G**) Summary table of affinity constants (* significant at *p* ≤ 0.01).

To elucidate the interaction between Lico A and *Tg*MORN1, we employed a combination of molecular docking and experimental validation (Figure 3A). Low-energy conformers of Lico A were generated and docked into a refined homology model of *Tg*MORN1, which revealed >95% residues within favored or allowed regions of the Ramachandran plot. SiteMap analysis identified ten potential binding cavities, three of which exhibited favorable binding free energies. Among these sites, Site 2 demonstrated the best steric complementarity and electrostatic distribution (Figure 3B), suggesting that it is the most plausible binding pocket. SPR confirmed the strong and specific binding of Lico A to *Tg*MORN1, with an affinity constant of ~15.3–15.6 nM (Figure 3A). To refine the interaction site, synthetic peptides corresponding to the three predicted binding regions were analyzed by SPR. Among them, the peptide corresponding to Site 2 exhibited the strongest binding response and the lowest dissociation constant (KD = 1.6 × 10^−6^ M), confirming that Site 2 was the dominant interaction site.

To pinpoint key residues within Site 2, five *Tg*MORN1 alanine mutants (R39A, T52A, S168A, D203A, D263A) were expressed in a cell-free system and evaluated by SPR across a concentration range of 10–2560 nM. The mutants R39A, T52A, and D263A retained affinities comparable to those of the wild type (K_D ~16–18 nM). In contrast, mutation of S168 resulted in a substantial loss of binding (K_D = 8.21 µM, ~536-fold reduction), whereas D203A almost completely abolished the interaction (K_D = 877 μM, > 57,000-fold reduction) (Figure 3D). These losses were accompanied by markedly reduced maximal binding responses (Figure 3D–G). Moreover, analysis of the binding curves (Figure 3E–F) together with affinity constants (Figure 3G) consistently demonstrated that residues S168 and D203 are indispensable for the recognition of *Tg*MORN1 by Lico A.

Sequence alignment revealed limited homology (<50% similarity) between *T. gondii* MORN1 and five human proteins, suggesting significant structural differences. Further analysis revealed that while the key binding residue S168 is present in some human homologs, D203 is absent from all human homologs. Additionally, notable gaps were observed in human homologs such as JP3, JPH4, and MORN1 (Figure S3 and Table S2).

### 3.5. Draggability Evaluation of Lico A

After systematic methodological validation, the PK profile of Lico A was characterized via liquid chromatography–tandem mass spectrometry (LC–MS/MS) following single oral or intraperitoneal administration (the results of methodological validation can be found in Tables S3–S11). Lico A exhibited satisfactory PK characteristics, suggesting potential suitability for oral delivery.

**Figure 4:**
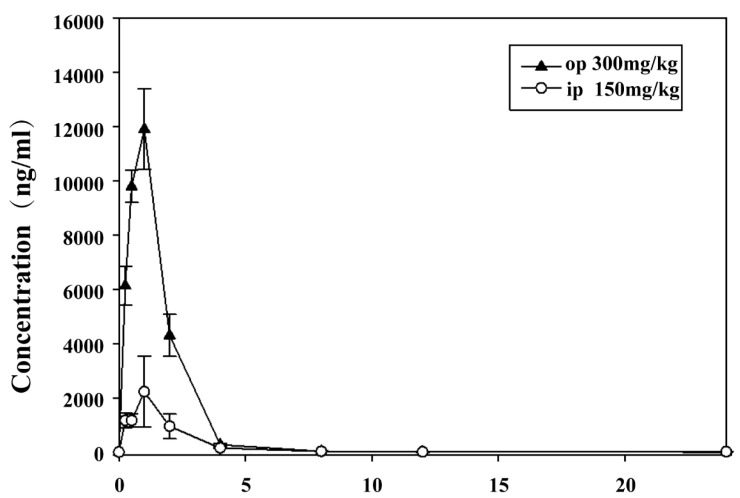
Plasma concentration–time curve. The two curves respectively represent the time-dependent changes in drug concentration after a single intraperitoneal injection of 150 mg/kg Lico A and a single oral administration of 300 mg/kg Lico A in female BALB/c mice, demonstrating that the drug has favorable in vivo absorption characteristics.

After a single intraperitoneal injection of 150 mg/kg Lico A in BALB/c female mice, the pharmacokinetic parameters were as follows: t_1/2_, 2.29 h; T_max_, 1.00 h; C_max_, 11,900 ng/mL; MRT_0–t_, 1.35 h; and AUC_0–t_, 21,600 ng·h/mL. Following a single oral gavage of 300 mg/kg, the corresponding values were as follows: t_1/2_, 1.96 h; T_max_, 1.00 h; C_max_, 2220 ng/mL; MRT_0–t_, 1.76 h; and AUC_0–t_, 4420 ng/h/mL. The drug–time curve is shown in Figure 4. The relative bioavailability (F) of i.p. versus oral administration was calculated to be 448.74%, suggesting that Lico A has potential for use in the development of oral and injectable anti-*T. gondii* drugs (Table S12).

To further assess whether Lico A can penetrate the blood–brain barrier (BBB), its brain distribution was evaluated in BALB/c mice following a single oral dose of 300 mg/kg. At 0.5, 1, and 8 h post-administration, the mean plasma concentrations were 1367, 1700, and 54.0 ng/mL, respectively, with an AUC of 7247 ng·h/mL and a dose-normalized AUC of 24.2 ng·h·kg/mL/mg. The corresponding brain concentrations were 1918, 2130, and 42.0 ng/g, yielding an AUC of 9094 ng·h/g and a dose-normalized AUC of 30.3 ng·h·kg/g/mg. At all three time points, the brain-to-plasma free drug concentration ratios (Kp, uu) were greater than 0.8, with a mean Kp, uu of 1.13. The dose-normalized AUC brain-to-plasma ratio (B/P) was 1.25 (Table S13).

### 3.6. Evaluation of the In Vitro Activity of the Toxoplasma gondii PRU Strain by Lico A

**Figure 5:**
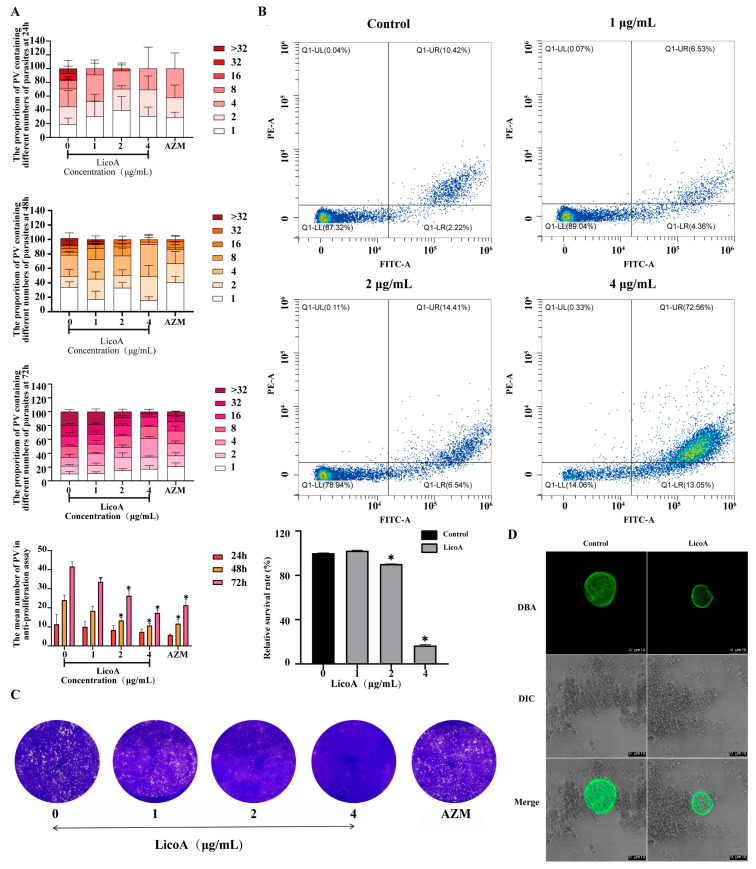
Evaluation of the in vitro activity of the *Toxoplasma gondii* PRU strain by Lico A. (**A**) Lico A decreased the number of PV and parasite number in PV of *Toxoplasma gondii* Pru strain after 24 h, 48 h, and 72 h incubation. (**B**) Flow cytometry analysis in Lico A induced death in *Toxoplasma gondii*. The survival of tachyzoites was analyzed by flow cytometry with treatment of 0 μg/mL, 1 μg/mL, 2 μg/mL, or 4 μg/mL Lico A for 24 h. (**C**) Plaques formed by *Toxoplasma gondii*-infected cells after Lico A treatment. Lico A (0, 1, 2, or 4 μg/mL) decreased *Toxoplasma gondii* growth inhibition in Vero cells. (**D**) Changes in the shape and size of *Toxoplasma gondii* PRU strain bradyzoite cyst after Lico A treatment. *Toxoplasma gondii* were treated with 2 μg/mL Lico A or without drug as a control for 48 h after infecting *Toxoplasma gondii* PRU strain tachyzoites (* significant at *p* ≤ 0.01).

Lico A significantly inhibited the proliferation ability of tachyzoites after 24 h, 48 h, and 72 h (*p* ≤ 0.01) (Figure 5). Compared to the control group, the number of parasitic vacuoles (PVs) after treatment with Lico A was significantly reduced (Figure 5A, *p* ≤ 0.01) in a time-dependent manner. Moreover, the number of tachyzoites per PV decreased in a dose-dependent manner, as shown in Figure 5A. Plaque formation assays demonstrated that both the area and number of plaques were reduced in a dose-dependent manner within the concentration range of 1–4 μg/mL, indicating that Lico A inhibited the proliferation of PRU strain tachyzoites (Figure 5C).

Flow cytometry was employed to assess the survival of Toxoplasma following Lico A treatment. As shown in Figure 5, treatment with 4 μg/mL Lico A significantly decreased these parameters (*p* < 0.01) (Figure 5B). The survival rate in the control group was approximately 87% (Figure 5B). Following 24 h of treatment with Lico A at concentrations of 1, 2, and 4 μg/mL, the survival rates of the parasites reduced, as presented in Figure 5B and 5D.

Compared to those of the control group, after Lico A incubation, the size of the cysts decreased, and the shape of the cysts changed significantly. These results indicated that Lico A could significantly inhibit the formation of cysts in *Toxoplasma gondii* PRU strains.

## 4. Discussion

The treatment of toxoplasmosis has long been hampered by a lack of effective drugs and by adverse effects, underscoring the importance of identifying novel targets and mechanisms for drug discovery. In this context, natural products have emerged as promising sources of antiparasitic agents, yet their mechanisms of action often remain elusive, hindering rational drug design and optimization. In this study, we established a multilayer, rigorously validated screening model that combines surface plasmon resonance–mass spectrometry (SPR-MS) with an artificial intelligence (AI)-driven multidimensional scoring system. Notably, this model was strengthened by multiple rounds of experimental verification, successfully demonstrating its ability to pinpoint Lico A’s antiparasitic target and to confirm the key interaction with TgMORN1. Through a series of complementary assays, we validated the direct binding of Lico A to *Tg*MORN1 and further elucidated the pharmacokinetic properties of Lico A. These findings have set an example for the targeted screening of natural products against toxoplasmosis, and more importantly, have highlighted the therapeutic potential of Lico A as a candidate drug for combating toxoplasmosis, which may ultimately contribute to the development of more effective and safer treatment options.

SPR-MS, as a label-free and real-time interaction analysis technology, allows direct measurement of the binding kinetics between candidate molecules and proteins, offering high sensitivity and quantitative advantages in target discovery [26]. AI, on the other hand, can leverage big data and deep learning to explore potential targets from multiple perspectives—including structural prediction, molecular docking, and network pharmacology—thus substantially improving the efficiency and breadth of target prediction [10,11]. Together, the precision of SPR and the predictive power of AI created a robust and reliable discovery pipeline, highlighting the accuracy and credibility of this integrated strategy. *Tg*MORN1 was shown to outperform other candidate targets in terms of affinity, enrichment, and network clustering; therefore, it is considered a particularly promising molecular target for Lico A.

Previous genetic studies have emphasized the indispensable role of MORN1 in *T. gondii*. Heaslip et al. [27] demonstrated that MORN1 is an essential component of the basal complex, with gene knockout leading to severe defects in cytokinesis, impaired parasite proliferation, and attenuated virulence. Similarly, Lorestani et al. [28] reported that MORN1 deficiency profoundly disrupts basal complex assembly, daughter cell budding, and apicoplast segregation, resulting in abnormal “double-headed” or multiheaded parasites. These findings underscore the pivotal function of MORN1 in parasite proliferation and provide a robust mechanistic rationale for its selection as a therapeutic target in this study.

RNAi-based gene silencing offers a rapid and robust strategy for the functional validation of putative targets. In this work, the silencing of *Tg*MORN1 significantly diminished the antiparasitic activity of Lico A, confirming that its efficacy is dependent on *Tg*MORN1. Additional assays, including Western blotting, NanoDSF, and SPR, corroborated the direct binding of Lico A to *Tg*MORN1 and its impact on protein thermostability. Site-directed mutagenesis further identified residues S168 and D203 within the MORN repeat domain as critical binding sites. Structural considerations suggest that S168 serves as a hydrogen bond donor, whereas the negative charge of D203 facilitates hydrogen bonding or electrostatic interactions. Together, these residues likely stabilize local conformations and promote ligand anchoring, thereby modulating the conformational dynamics and function of *Tg*MORN1—findings that align with the essential role of MORN1 in basal complex assembly and parasite cell division.

Pharmacokinetic studies have previously reported a low absolute oral bioavailability of Lico A (3.3%) in rats [29], indicating its limited absorption but potential for oral delivery. In this study, we compared oral and intraperitoneal administration, 300 mg/kg and 150 mg/kg, respectively [24], and the results revealed that intraperitoneal dosing achieved a remarkably high relative bioavailability (448.74%), eliminating gastrointestinal absorption barriers and providing a more accurate assessment of systemic exposure. Encouragingly, our data revealed excellent blood–brain barrier (BBB) penetration by Lico A, with substantial brain exposure (Kp,uu = 1.13, B/P = 1.25). BBB permeability is commonly evaluated by the brain–plasma concentration ratio (B/P) and the ratio of unbound drug in the brain to that in the plasma (Kp,uu), where B/P > 0.3 or a Kp, uu value close to 1 indicates favorable brain distribution [30]. This implies that Lico A is able to reach sites of latent cerebral infection. This finding is particularly important because *T. gondii* initially forms latent brain cysts, which then lead to chronic infection; most current therapies are ineffective against these cyst forms owing to poor BBB permeability and limited intracerebral efficacy. Together with its antiparasitic potency, its favorable brain distribution supports the view that Lico A may serve as a promising candidate for chronic cerebral toxoplasmosis. The optimization of formulations, such as nanocarriers or solid dispersions, could further enhance their oral performance and should be examined in future studies.

*T. gondii* brain cysts, caused by *Toxoplasma gondii*, are the main factor responsible for long-term latency; these cysts enable transmission and are also a key reason why chronic infections are difficult to cure [31]. Current clinical first-line drugs, such as sulfadiazine and pyrimethamine, mainly target the actively dividing tachyzoite stage but have little effect on cysts in a “dormant” state. This study revealed that Lico A can not only kill tachyzoites but also significantly inhibit cyst formation in the PRU strain and induce morphological changes, thereby blocking the establishment and maintenance of chronic infection. This result has important scientific innovation and clinical transformation prospects. Notably, this property may give it a unique advantage in the brain and other sites where cysts are prone to form and difficult to eliminate. Therefore, this study provides direct evidence for the development of Lico A as an anti-*Toxoplasma* drug and, more importantly, a new research direction and therapeutic strategy for solving the clinical problem of chronic *Toxoplasma* infection.

Despite these advances, several issues remain to be addressed. First, while our dsRNA soaking experiments provided strong evidence for the role of *Tg*MORN1 in Lico A, certain methodological limitations should be considered. This method relies on the parasite’s endogenous uptake mechanisms, which may not be uniformly efficient across the entire population and typically result in partial rather than complete knockdown. We opted for this method to preserve high parasite viability, which was crucial for subsequent phenotypic assays (e.g., *Toxoplasma gondii* tachyzoite proliferation). While the observed knockdown level (approximately 70% reduction in mRNA, as confirmed by qRT–PCR) was sufficient to elicit a reproducible phenotypic effect that mirrored Lico A treatment, we acknowledge that variable silencing efficiency is a constraint of this technique. Although we designed specific dsRNAs, the potential for off-target effects (OTEs) cannot be entirely ruled out. OTEs occur when dsRNA silences genes with partial sequence homology, leading to misinterpretation of the phenotype. In our case, the concordance between the Lico A-induced phenotype and the dsRNA knockdown phenotype strongly suggests the observed effects are target-specific. Nevertheless, future studies employing complementary approaches, such as conditional knockout or CRISPR interference (CRISPRi), would be valuable to confirm these findings with higher specificity. Second, although *Tg*MORN1 has been confirmed as the primary target of Lico A, potential synergistic interactions with additional molecular targets cannot be ruled out, warranting more comprehensive omics and functional studies. Third, while our sequence alignment reveals homology between the *T. gondii* target and human MORN-like proteins, a structural comparison would offer more definitive insights into functional conservation and potential drug selectivity. An analysis of predicted structures suggests that while the core MORN-fold might be conserved, key divergences likely exist in loop regions, surface electrostatics, or the topology of potential ligand-binding pockets. These structural differences could underlie the observed anti-parasitic efficacy of Lico A without significant cytotoxicity to human cells, meriting further investigation. Future work employing computational approaches such as comparative modeling, molecular docking of Lico A, and molecular dynamics simulations, followed by experimental validation against the human homologs, will be essential to confirm this hypothesis of selectivity and guide the rational development of specific anti-Toxoplasma agents. Finally, while pharmacokinetic data indicate effective brain accumulation of Lico A and in vitro assays demonstrate its ability to disrupt *Toxoplasma gondii* cyst formation, its sustained and potent antiparasitic efficacy against chronic infection in vivo remains to be conclusively demonstrated through rigorous in vivo therapeutic experiments. Although previous studies have shown that Lico A is mainly metabolized through phase I oxidation and phase II binding reactions, among which glucuronidation is its main clearance pathway (Figures S4 and S5), the exposure of its metabolites in the central nervous system and their potential off-target binding effects still require further research. Since Lico A is being developed as a therapeutic drug for chronic *Toxoplasma gondii* infection, a comprehensive assessment of these factors is crucial for ensuring its safety and efficacy.

## 5. Conclusions

In summary, this study demonstrated that *Tg*MORN1 is the direct molecular target of Lico A, revealing its favorable in vivo absorption properties–particularly its high blood—brain barrier (BBB) permeability—and confirming its ability to disrupt the cyst formation of *Toxoplasma gondii* PRU strains. These findings underscore its potential as a promising therapeutic candidate for toxoplasmosis, especially in cases involving chronic cerebral infection, which are notoriously difficult to treat.

## Data Availability

The original contributions presented in this study are included in the article/Supplementary Materials. Further inquiries can be directed to the corresponding authors.

## Supplementary Materials

The following supporting information can be downloaded at: https://www.mdpi.com/article/10.3390/biom16030410/s1, Figure S1: Overview of algorithm and model files; Figure S2: Results of affinity purification of serum antibodies; Figure S3: Comparison result between *Toxoplasma gondii* protein (MORN1) and Human protein (MORN1); Figure S4: Diagrams of possible phase I metabolic pathways of Lico A in liver microsomes of mice, cats, beagles, cattle, pigs, cynomolgus monkeys and humans; Figure S5: Diagrams of possible phase II metabolic pathways of Lico A in liver microsomes of mice, cats, beagles, cattle, pigs, cynomolgus monkeys and humans; Figure S6: Original Western blotting image; Table S1: The full-length gene of the target protein; Table S2: Comparison between *Toxoplasma gondii* protein and human protein; Table S3: Applicability of the plasma system; Table S4: Applicability of the brain tissue system; Table S5: Results of the precision and accuracy of plasma sample; Table S6: Results of the precision and accuracy of brain tissue; Table S7: Recovery results of plasma sample; Table S8: Recovery results of brain tissue; Table S9: Results of plasma sample stability; Table S10: Whole blood stability results; Table S11: Results of brain tissue sample stability; Table S12: Pharmacokinetic parameters of balb/c mice after intraperitoneal injection and intragastric administration of Lico A; Table S13: The brain tissue distribution results of Balb/c female mice after intragastric administration of 300 mg/kg LicoA.
