# Supplementary material for: Licochalcone A as a Potential Anti-Toxoplasma Agent: A Target Identification and Pharmacokinetic Study
Source: Biomolecules. 2026 Mar 10;16(3):410. doi: 10.3390/biom16030410 (PMC13024206; doi:10.3390/biom16030410)
Supplement: Supplementary file 1 [file biomolecules-16-00410-s001.zip › biomolecules-4137192-supplementary.pdf]

biomolecules

Figures

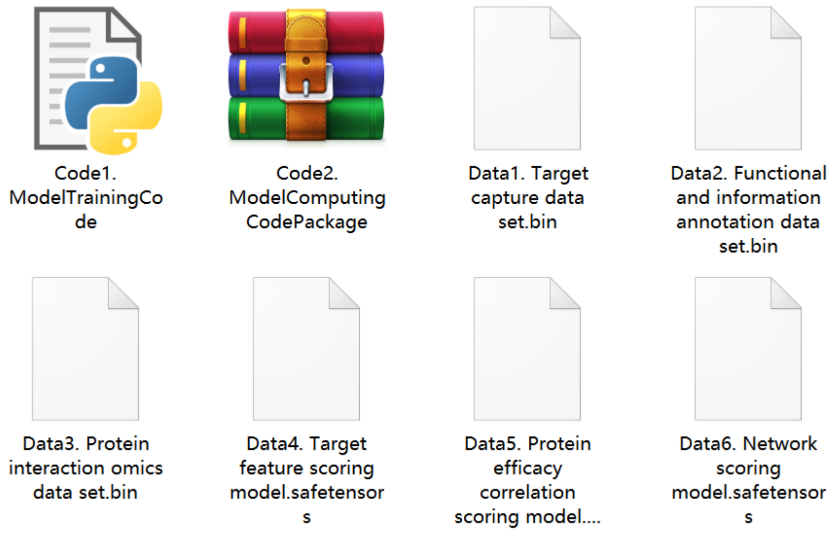

Figure S1. Overview of Algorithm and Model Files.

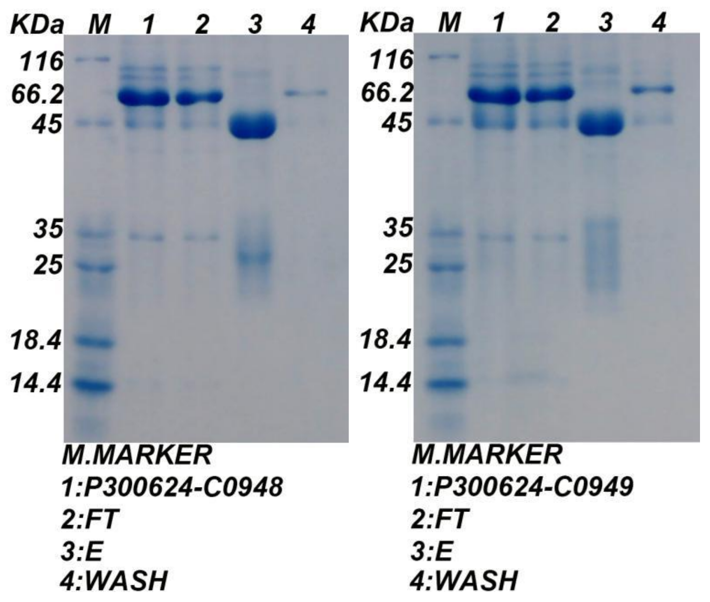

Figure S2. Results of affinity purification of serum antibodies.

```
Query_10001  1    MESCHAYHGQIKDGLFHGKGTLIYSGNEKYEGEFVFGKREGHGRFLYADGATYEGKWVEDRIHGQGVAHFASGNRYEGQW  80
Query_10002  1    MAAA-------------GEGTPSSRGPRRDPPRRP--PRNGYGVYVYP--------------------------------  33

Query_10001  81   EMGRINGFGKLSYSNGDEYEGEWVDGKMHGRGTYRYAEGDVYTGEWRDDKRHGKGSVTYVSAKGSVVEKYEGDWVNGKMH  160
Query_10002  34   -----NSFFR--------YEGEWKAGRKHGHGKLLFKDGSY----------------------------YEGAFVDGEIT  72

Query_10001  161  GHGKYIYS-DGGVYEGDWIDGKMHGKGTYVFPNGNVYEGEWAHDMKDGYGVLTYQNGEKYEGYWKQDKVHGKGTLTYTRG  239
Query_10002  73   GEGRRHWAWSGDTFSGQFVLGEPQGYGVMEYKAGGCYEGEVSHGMREGHGFLVDRDGQVYQGSFHDNKRHGPGQMLFQNG  152

Query_10001  240  DKYIGDWMDAKKDGEGELIYANGDRFKGQWADDRANGFGVFTYANGNRYEGEWTDD---KRHGRGVFYCAE-----DGSA  311
Query_10002  153  DKYDGDWVRDRRQGHGVLRCADGSTYKGQWHSDVFSGLGSMAHCSGVTYYGLWINGHPAEQATRIVILGPEVMEVAQGSP  232

Query_10001  312  YE---------GEFVGGRKEGNGILRLATGHQLE--GTWSGGQLVRV----------TSFVFA-----------------  353
Query_10002  233  FSVNVQLLQDHGEIA--KSESGRVLQISAGVRYVQLSAYSEVNFFKVDRDNQETLIQTPFGFECIPYPVSSPAAGVPGPR  310

Query_10001  354  -------QDSPWLNVDL-----------------------  363
Query_10002  311  AAKGGAEADVPLPRGDLELYLGALHGQEDTPGGLLGSSLF  350
```

Figure S3. Comparison result between *Toxoplasma gondii* protein(MORN1)) and Human protein (MORN1)

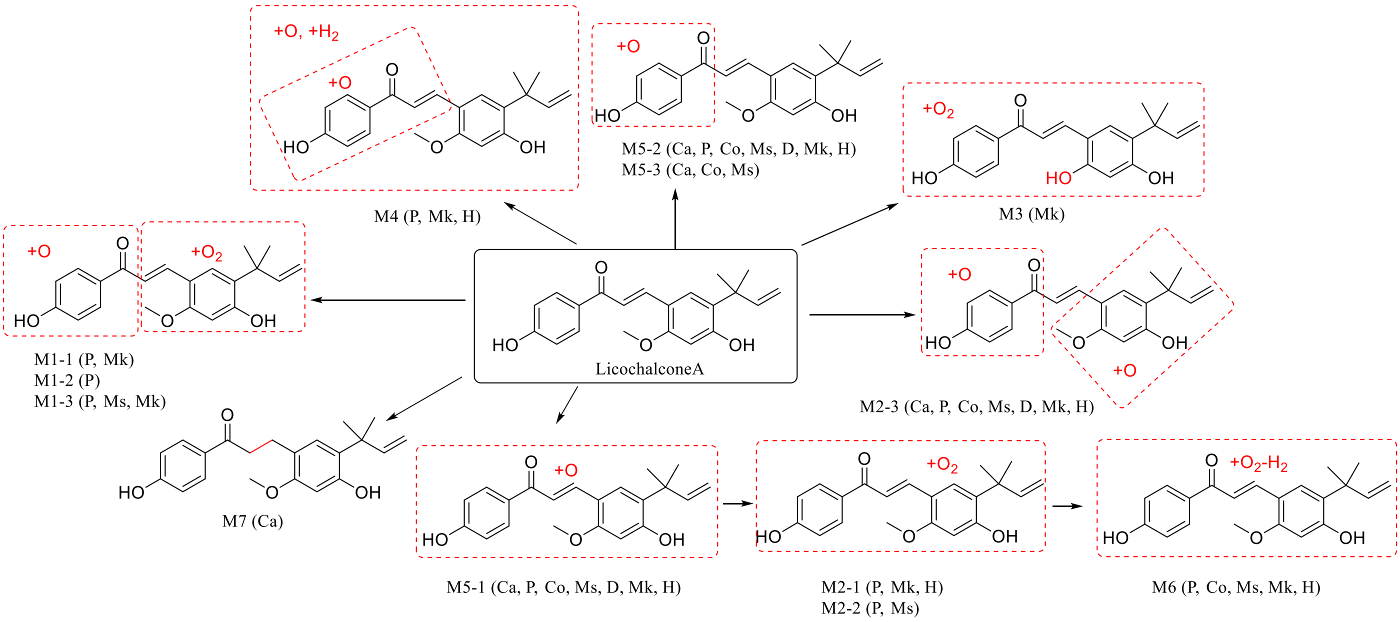

Figure S4. Diagrams of possible phase I metabolic pathways of Lico A in liver microsomes of mice, cats, beagles, cattle, pigs, cynomolgus monkeys and humans Ca: Cat liver microsomal P: Pig liver microsomal Co: Bovine liver microsomal Ms: mouse liver microsomal D: Dog liver microsomal Mk: monkey liver microsomal H: Human liver microsomal.

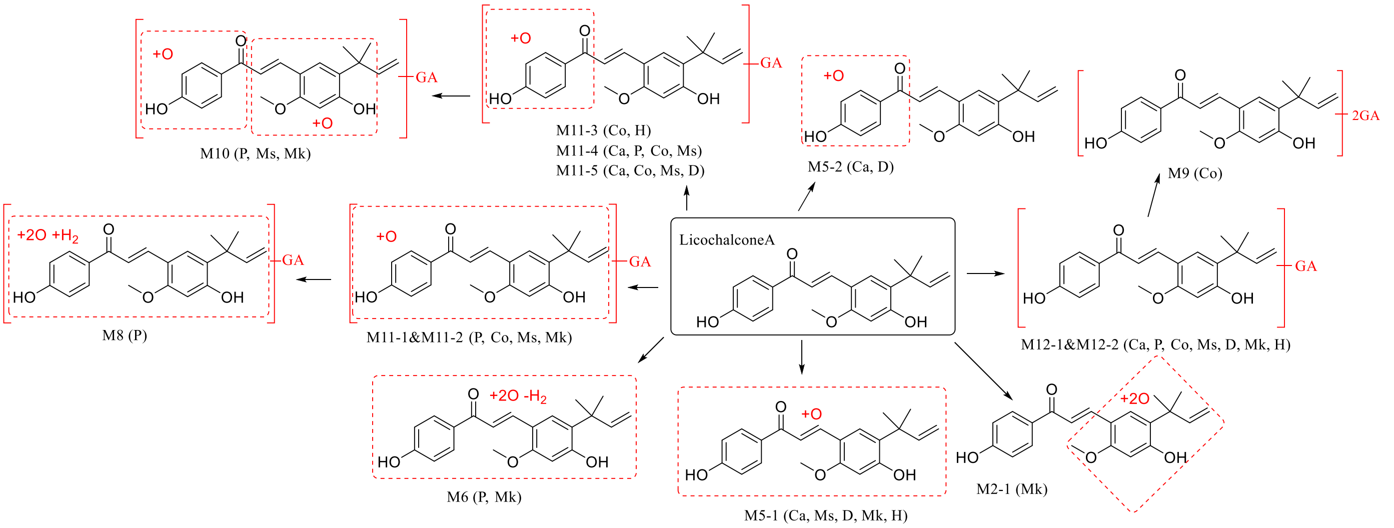

Figure S5. Diagrams of possible phase Ⅱ metabolic pathways of Lico A in liver microsomes of mice, cats, beagles, cattle, pigs, cynomolgus monkeys and humans Ca: Cat liver microsomal P: Pig liver microsomal Co: Bovine liver microsomal Ms: mouse liver microsomal D: Dog liver microsomal Mk: monkey liver microsomal H: Human liver microsomal.

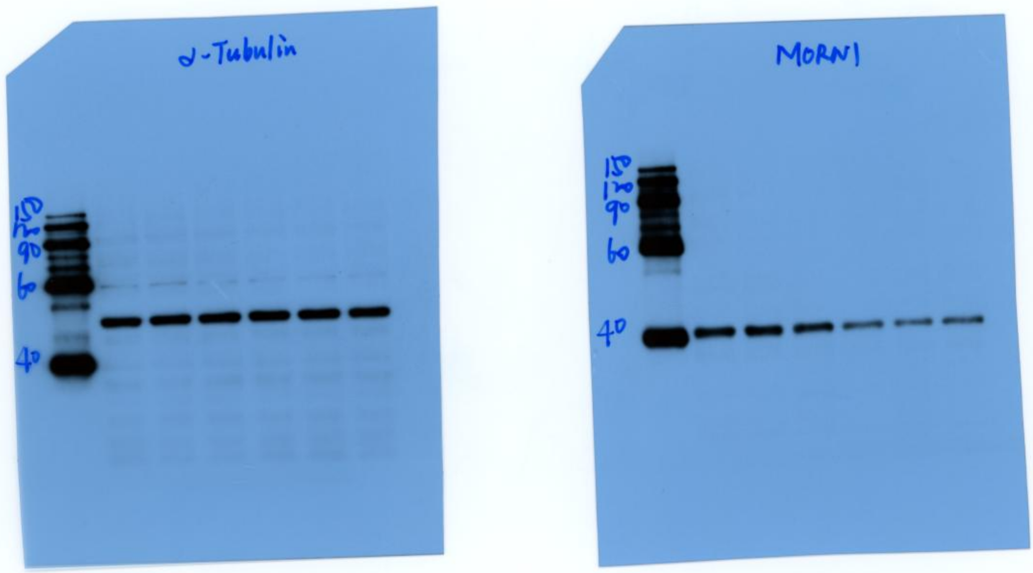

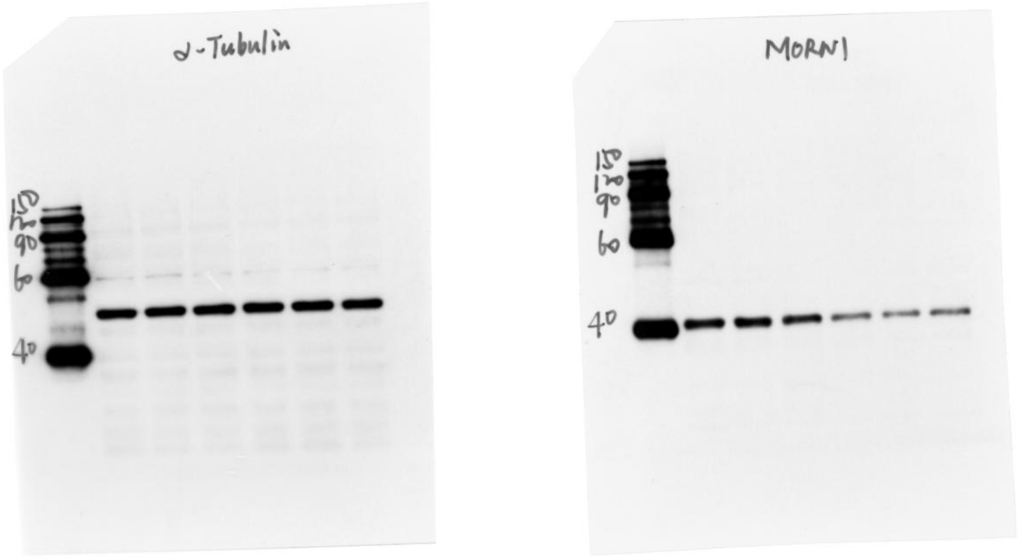

Figure S6. Original Western blotting image.

Tables

Table S1. The full-length gene of the target protein.

Full-length gene of *Tg*MORN1
MESCHAYHGQIKDGLFHGKGTLIYSGNEKYEGEFVFGKREGHGRFLYADGATYEGKWVEDRI-
HGQGVAHFASGNRYEGQWEMGRINGFGKLSYSNGDEYEGEWVDGKMHGRGTYRYAEGDVYTGEWRDDKRHGKGS
VTYVSAKGSVVEKYEGDWVNGKMHGHGKY-
IYSDGGVYEGDWIDGKMHGKGTYVFPNGNVYEGEWAHDMKDGYGVLTYQNGEKYEGYWKQDKVHGKGTLTYTRG
DKYIGDWMDAKKDGEGELIYANG-
DRFKGQWADDRANGFGVFTYANGNRYEGEWTDDKRHGRGVFYCAEDGSAYEGEFVGGRKEGNGILRLATGHQLEGT
WSGGQLVRVTSFVFAQDSPWLNVDLLEHHHHHH*

Table S2. Comparison between Toxoplasma gondii protein and human protein.

Toxoplasma gondii protein(MORN1)	Human protein(MORN1)	Details of *Toxoplasma gondii* protein
A0A2T6J2T7	AAH21704.1	Membrane occupation and recognition nexus protein MORN1 OS=*Toxoplasma gondii* GN=TGBR9_310440 PE=1 SV=1 - [A0A2T6J2T7_TOXGO]

Table S3. Applicability of the plasma system.

Sample name	LicoA Retention time（min）	IR Retention time（min）	Peak area ratio
LicoA-1	1.37	1.43	0.00945
LicoA-2	1.37	1.42	0.00982
LicoA-3	1.37	1.43	0.0101
LicoA-4	1.37	1.43	0.0102
LicoA-5	1.36	1.42	0.0102
LicoA-6	1.37	1.43	0.00835
Mean	1.37	1.43	0.00969
SD	0.00408	0.00516	0.000715
RSD	0.3	0.4	7.4

Table S4. Applicability of the brain tissue system.

Sample name	LicoA Retention time（min）	IR Retention time（min）	Peak area ratio
LicoA-1	1.52	1.44	0.0417
LicoA-2	1.51	1.43	0.0451
LicoA-3	1.51	1.43	0.0421
LicoA-4	1.50	1.42	0.0448
LicoA-5	1.51	1.42	0.0431
LicoA-6	1.51	1.43	0.0454
Mean	1.51	1.43	0.0437
SD	0.00632	0.00753	0.00161
RSD	0.4	0.5	3.7

Table S5. Results of the precision and accuracy of plasma sample.

QCs	LOQ	LQC	MQC	HQC
Theoretical concentration（ng/mL）	5	15	1500	4000
Sample name	concentration（ng/mL）			
LicoA-1	4.61	14.8	1300	4170
LicoA-2	4.40	14.3	1300	4160
LicoA-3	4.36	14.5	1330	4160
LicoA-4	4.61	14.7	1300	4180
LicoA-5	4.27	14.9	1260	4130
LicoA-6	4.20	15.4	1280	4240
Mean（ng/mL）	4.41	14.8	1295	4173
SD	0.171	0.378	23.5	36.7
RSD	3.9	2.6	1.8	0.9
RE%	-11.8	-1.6	-13.7	4.3
n	6	6	6	6

Table S6. Results of the precision and accuracy of brain tissue.

QCs	LOQ	LQC	MQC	HQC
Theoretical concentration （ng/mL）	5	15	1500	4000
Sample name	concentration（ng/mL）			
LicoA-1	1.12	2.79	80.0	737
LicoA-2	0.858	2.69	78.3	719
LicoA-3	0.997	2.66	83.0	723
LicoA-4	1.09	2.47	75.5	732
LicoA-5	1.12	2.82	78.0	771
LicoA-6	0.965	2.71	79.0	737
Mean（ng/mL）	1.03	2.69	79.0	737
SD	0.104	0.124	2.48	18.4
RSD	10.2	4.6	3.1	2.5
RE%	2.5	-10.3	-1.3	-7.9
n	6	6	6	6

Table S7. Recovery results of plasma sample.

Sample name	Theoretical concentration（ng/mL）	Peak area ratio	Recovery（%）
LicoA L-1	15	0.0326	97.7
LicoA L-2		0.0312	93.5
LicoA L-3		0.0320	95.9
	Mean		95.7
	SD		2.11
	RSD		2.2
LicoA M-1	1500	2.96	102
LicoA M-2		2.97	102
LicoA M-3		2.93	101
	Mean		101
	SD		0.715
	RSD		0.7
LicoA H-1	4000	8.84	98.6
LicoA H-2		8.96	99.9
LicoA H-3		9.10	101
	Mean		100
	SD		1.45
	RSD		1.5
		n	9
		Mean	99.0
		SD	2.88
		RSD	2.9

Table S8. Recovery results of brain tissue.

Sample name	Theoretical concentration（ng/mL）	Peak area ratio	Recovery（%）
LicoA L-1	3	0.0930	106
LicoA L-2		0.0858	97.9
LicoA L-3		0.0871	99.4
	均值		101
	SD		4.38
	RSD		4.3
LicoA M-1	80	2.54	106
LicoA M-2		2.48	104
LicoA M-3		2.48	104
	Mean		105
	SD		1.45
	RSD		1.4
LicoA H-1	800	23.4	104
LicoA H-2		23.4	104
LicoA H-3		22.3	99.4
	Mean		103
	SD		2.83
	RSD		2.8
		n	9
		Mean	103
		SD	3.10
		RSD	3.0

Table S9. Results of plasma sample stability.

QCs		LQC	HQC
Theoretical concentration （ng/mL）		15	4000
Stability type<br>Experimental conditions/period	Sample number	concentration（ng/mL）	
Stability of the laboratory bench<br>Room temperature /0h	LicoA-1	16.8	4370
	LicoA-2	15.8	4270
	LicoA-3	15.7	4310
	LicoA-4	15.7	4330
	LicoA-5	16.2	4350
	LicoA-6	16.0	4340
	Mean（ng/mL）	16.0	4328
	SD	0.423	34.9
	RSD	2.6	0.8
	%RE	6.9	8.2
	n	6	6
Stability of the laboratory bench<br>Room temperature /6h	LicoA-1	16.4	4000
	LicoA-2	14.7	4020
	LicoA-3	16.1	4070
	LicoA-4	18.0	4030
	LicoA-5	14.0	4020
	LicoA-6	16.4	4000
	均值（ng/mL）	15.9	4023
	SD	1.41	25.8
	RSD	8.9	0.6
	%RE	6.2	0.6
	n	6	6
Stability of the injector<br>15°C/48h	LicoA-1	16.0	4260
	LicoA-2	14.8	4150
	LicoA-3	15.9	4310
	LicoA-4	15.1	4160
	LicoA-5	15.3	4260
	LicoA-6	15.4	4300
	Mean（ng/mL）	15.4	4240
	SD	0.462	69.0
	RSD	3.0	1.6
	%RE	2.8	6.0
	n	6	6

Table S10. Whole blood stability results.

Sample name	Experi-mental con-ditions	Placement time（h）	Peak area ratio	Mean	RSD	RD%
LicoA-L-1-0h	wet ice	0	0.0242	0.0243	3.2	NA
LicoA-L-2-0h			0.0228			
LicoA-L-3-0h			0.0242			
LicoA-L-4-0h			0.0247			
LicoA-L-5-0h			0.0249			
LicoA-6-0h			0.0248			
LicoA-L-1-1h		1	0.0246	0.0250	2.6	3.0
LicoA-L-2-1h			0.0255			
LicoA-L-3-1h			0.0241			
LicoA-L-4-1h			0.0250			
LicoA-L-5-1h			0.0259			
LicoA-L-6-1h			0.0248			
LicoA-H-1-0h	wet ice	0	6.66	6.68	1.1	NA
LicoA-H-2-0h			6.68			
LicoA-H-3-0h			6.69			
LicoA-H-4-0h			6.66			
LicoA-H-5-0h			6.81			
LicoA-H-6-0h			6.58			
LicoA-H-1-1h		1	6.72	6.76	1.3	1.2
LicoA-H-2-1h			6.83			
LicoA-H-3-1h			6.62			
LicoA-H-4-1h			6.85			
LicoA-H-5-1h			6.74			
LicoA-H-6-1h			6.81			

Table S11. Results of brain tissue sample stability.

QCs		LQC	HQC
Theoretical concentration （ng/mL）		15	4000
Stability type<br><br>Experimental conditions/period	Sample number	concentration（ng/mL）	
Stability of the laboratory bench<br><br>Room temperature /0h	LicoA-1	3.21	759
	LicoA-2	2.84	739
	LicoA-3	2.83	719
	LicoA-4	3.02	759
	LicoA-5	2.97	768
	LicoA-6	2.96	750
	Mean（ng/mL）	2.97	749
	SD	0.139	17.7
	RSD	4.7	2.4
	%RE	-0.9	-6.4
	n	6	6
Stability of the laboratory bench<br><br>Room temperature /6h	LicoA-1	3.14	904
	LicoA-2	2.94	838
	LicoA-3	3.16	800
	LicoA-4	3.01	824
	LicoA-5	3.13	828
	LicoA-6	3.00	855
	Mean（ng/mL）	3.06	842
	SD	0.0914	35.5
	RSD	3.0	4.2
	%RE	2.1	5.2
	n	6	6
Stability of the injector 15°C/48h	LicoA-1	2.73	754
	LicoA-2	3.02	729
	LicoA-3	2.71	744
	LicoA-4	2.52	751
	LicoA-5	2.72	801
	LicoA-6	3.05	768
	Mean（ng/mL）	2.79	758
	SD	0.204	24.7
	RSD	7.3	3.3
	%RE	-6.9	-5.3
	n	6	6

Table S12. Pharmacokinetic parameters of balb/c mice after intraperitoneal injection and intragastric administration of Lico A.

PK Parameter	Unit	LicoA	
		Group A 150 mg/kg	Group B 300 mg/kg
		Mean	Mean
Kel	1/h	0.30	0.35
$t_{1/2}$	h	2.29	1.96
T_{max}	h	1.00	1.00
C_{max}	ng/mL	11900	2220
$AUC_{0\text{-}t}$	ng·h/mL	21600	4420
$AUC_{0\text{-}inf}$	ng·h/mL	21700	4440
$MRT_{0\text{-}t}$	h	1.35	1.76
$MRT_{0\text{-}inf}$	h	1.40	1.83
F	%	488.74%	-

Table S13. The brain tissue distribution results of Balb/c female mice after intragastric administration of 300 mg/kg LicoA.

Sample	Dose mg/kg	Time	Brain concentration (ng/g)				Plasma concentration (ng/mL)				Brain/plasma concentration ratio				AUCall		Dose normalization AUCall		
	administration	(h)	1	2	3	Mean	1	2	3	Mean	1	2	3	Mean	AUC Brain / ng·h/g	AUC Plasma ng·h/mL	AUC Brain ng·h·kg/g/mg	AUC plasma ng·h·kg/mL/mg	Ratio
Lico A	p.o.	0.5	1380	3768	606	1918	1080	2260	760	1367	1.28	1.67	0.78	1.25	9094	7247	30.30	24.20	1.25
		1	3096	1698	1596	2130	1850	2240	1010	1700	1.67	0.76	1.58	1.34					
	300	8	53.10	21.20	51.70	42.00	83.00	30.40	48.50	54.00	0.64	0.70	1.07	0.80					
